# Fracture-related infections after osteosynthesis for hip fracture are associated with higher mortality: A retrospective single-center cohort study

**DOI:** 10.2340/17453674.2024.41980

**Published:** 2024-09-23

**Authors:** Pendar KHALILI, Anders BRÜGGEMANN, Staffan TEVELL, Per FISCHER, Nils P HAILER, Olof WOLF

**Affiliations:** 1 Department of Surgical Sciences, Orthopaedics, Uppsala University, Uppsala; 2 Department of Orthopedic Surgery, Karlstad Hospital, Karlstad; 3 Centre for Clinical Research and Education, Region Värmland, Karlstad; 4 Department of Infectious Diseases, Karlstad Hospital, Karlstad; 5 School of Medical Sciences, Faculty of Medicine and Health, Örebro University, Örebro, Sweden

## Abstract

**Background and purpose:**

Fracture-related infections (FRIs) after osteosynthesis for hip fractures have not been thoroughly investigated. Our primary aim was to assess the association between FRIs and mortality after osteosynthesis for hip fracture. Secondary aims were to investigate the incidence, microbiology, and general epidemiological aspects of these FRIs.

**Methods:**

This retrospective single-center study included 1,455 patients > 18 years old with non-pathological hip fractures treated with osteosynthesis between 2015 and 2019. Medical records were reviewed and FRIs were diagnosed based on current consensus criteria. The follow-up period was 2 years. Mortality was estimated using Kaplan–Meier survival analysis. Cox regression analyses were performed to investigate the potential association between FRIs, as a time-dependent variable, and increased mortality.

**Results:**

The median age for the entire cohort was 83 (interquartile range 75–89) years and 69% were females. At the 2-year follow-up mark, the crude mortality rate was 33% in the non-FRI group and 69% (11 of 16 patients) in the FRI group. Cox regression analysis assessing mortality risk revealed a hazard ratio of 3.5 (95% confidence interval [CI] 1.9–6.4) when adjusted for confounders. The incidence of FRI was 1.1% (16 of 1,455 patients). *Staphylococcus aureus* was the most common pathogen. Most FRI patients (94%) required at least 1 revision and 56% underwent ≥ 2 revision.

**Conclusion:**

We found an association between FRIs after hip fracture osteosynthesis and increased mortality, underscoring the critical need for FRI prevention measures in this frail patient group. The incidence and microbiological findings were consistent with previous studies.

Hip fractures are a serious health concern, leading to significant limitations in physical activity and increased mortality [1]. These fractures often affect frail patients with multiple comorbidities, rendering recovery a complex and challenging process [2]. Fracture site infection after surgery could exacerbate this challenge, creating further hurdles in the patient’s path to recovery.

The incidence of deep infection after hip fracture surgery is reported to be between 0.7% and 2.5% [3-6]. These infections can be devastating, often requiring multiple surgical interventions, extended courses of antibiotics, longer hospital stays, and more intensive rehabilitation [4,5]. The burden extends beyond the patient, affecting the healthcare systems with increased costs and needs [3,6]. Postoperative infection at the fracture site is a broad concept, very often not well defined in the literature, which may lead to inconsistent results and conclusions [7]. The definition of fracture-related infection (FRI), established by an international panel of experts in 2018, is designed to aid in diagnosis and standardize terminology across the literature [8].

Understanding when an FRI typically occurs, which pathogens are most common, and how to adapt treatment is crucial for improving postoperative patient care. Previous studies analyzing postoperative infection at the fracture site after hip fracture surgery often lack consistency in defining FRI, or use mixed cohorts consisting of FRIs after osteosynthesis surgery as well as prosthetic joint infections (PJIs) [4,9-11]. Wong et al. observed that an FRI was associated with increased mortality in a cohort that comprised 311 fractures, mainly in the femur and tibia. However, their research did not specifically address the impact of FRIs on hip fractures after osteosynthesis [12].

Our primary aim was to assess the association between FRIs and mortality following osteosynthesis for hip fracture, excluding arthroplasties and thus PJIs. Secondary aims were to describe the general epidemiological aspects of the typical hip FRI patient in terms of age, sex, and comorbidity.

## Methods

### Study design

This single-center, retrospective cohort study investigated patients over 18 years of age treated for hip fractures at a tertiary center (Uppsala University Hospital, Sweden) during 2015–2019. ICD-10 codes were used to identify patients with hip fractures, specifically S72.0 (Neck of femur fracture), S72.1 (Trochanteric fracture), and S72.2 (Subtrochanteric fracture). Surgical treatment was identified through the Nordic Medico-Statistical Committee (NOMESCO) procedure codes, and all arthroplasties were excluded. Further exclusion criteria were patients with pathological fractures and patients where treatment, postoperative care, or follow-up was performed at another unit. The follow-up time was set at 2 years after index surgery. The study is reported according to STROBE guidelines.

### Data collection and variables

Data was obtained from medical records and the Swedish Fracture Register (SFR), a national quality register established in 2012 [13]. The SFR, linked to all Swedish orthopedic departments, includes information on injury mechanisms, fracture types, and treatments. For this study, data from the SFR was extracted specifically for all hip fracture patients from this tertiary center. Variables and complications were reviewed to complete the dataset, and additional details were extracted for patients with FRIs by reviewing medical records. Patient information encompassed age, date of injury (from the SFR), type of hip fracture (identified by ICD-10 codes and the SFR), date and surgical treatment; intramedullary nail, sliding hip screw, screw/pin fixation (typically 2 cannulated screws or pins), or other combined (typically sliding hip screw in combination with extra screw/pin) (identified by NOMESCO codes and the SFR), the American Society of Anesthesiologists (ASA) classification, sex, presence of dementia, and date of death. If antibiotic prophylaxis was administered, it was given intravenously approximately 15–30 minutes before the procedure, with the routine protocol being either 2 grams of cloxacillin given 3 times (at 0, 2, and 8 hours) or 600 mg of clindamycin given twice (at 0 and 8 hours) for patients with penicillin allergy. Minor variations in the protocol occurred due to factors such as skin condition, choice of osteosynthesis, and other clinical circumstances.

This study considered only first-time FRI episodes, excluding relapses or recurrences. The time to FRI onset was defined as the interval from primary osteosynthesis to the initial appearance of symptoms according to FRI criteria. Specific variables for patients with FRIs included causative pathogens, reoperations for infection and all other causes, and antibiotic treatment, including information on which antibiotics were used and for how long. Removal of hardware was also classified as a revision surgical procedure. All patients were censored for further revision surgery, loss to follow-up, or end of 2-year follow-up, whichever came first. The SFR has been validated for femoral fractures, demonstrating agreement between recorded classifications and a gold standard [14].

### Initial cohort screening

**Figure 1:**
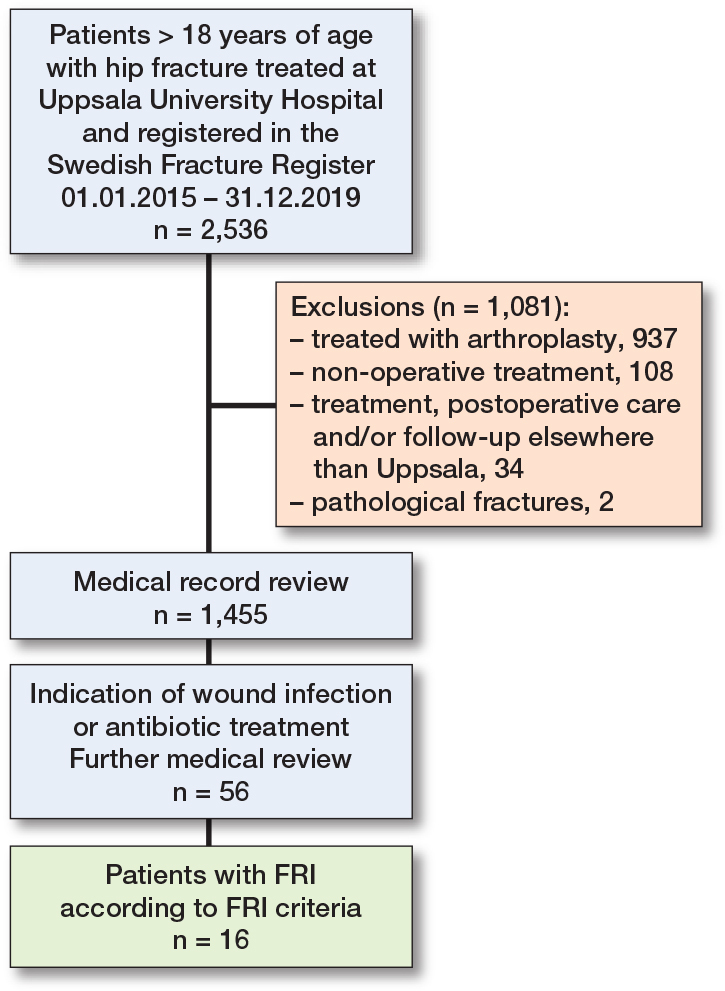
Flowchart illustrating patient selection.

All medical records from the initial cohort of patients with hip fractures were reviewed. Patients were excluded if they had undergone hemiarthroplasty, total hip arthroplasty, had pathological fractures, received non-surgical treatment, or if their surgical treatment, postoperative care and/or follow-up had not been conducted in Uppsala. Thus, a subset of patients was identified, and from this subset the specific FRI group was subsequently identified based on signs of postoperative infectious complications, such as wound complications/infection or extended postoperative antibiotic use (Figure 1). All information was found in the medical records. Routine follow-up of hip fracture patients is not conducted at the tertiary center, but it does occur to some extent among junior operating colleagues who need to gather clinical experience, as well as for certain patients who, for various reasons, such as complications pre-, peri-, or postoperatively, needed follow-up.

### FRI group

The FRI group included patients with FRIs according to the definition based on the updated consensus published by Govaert et al. in 2020 [15]. Confirmatory criteria were (i) phenotypically indistinguishable pathogens identified by culture from at least 2 separate deep tissue/implant specimens taken during an operative intervention (including sonication fluid), or (ii) fistula, sinus, or wound breakdown with communication to the bone or the implant, or (iii) purulent drainage from the wound or presence of pus during surgery. Confirmatory criteria also include (iv) the presence of microorganisms in deep tissue specimens, confirmed by histopathological examination, or (v) the presence of ≥ 5 polymorphonuclear neutrophils per high-power field, confirmed by histopathological examination. However, during the study period, histopathology was not performed at the studied tertiary center.

### Statistics

**Figure 2:**
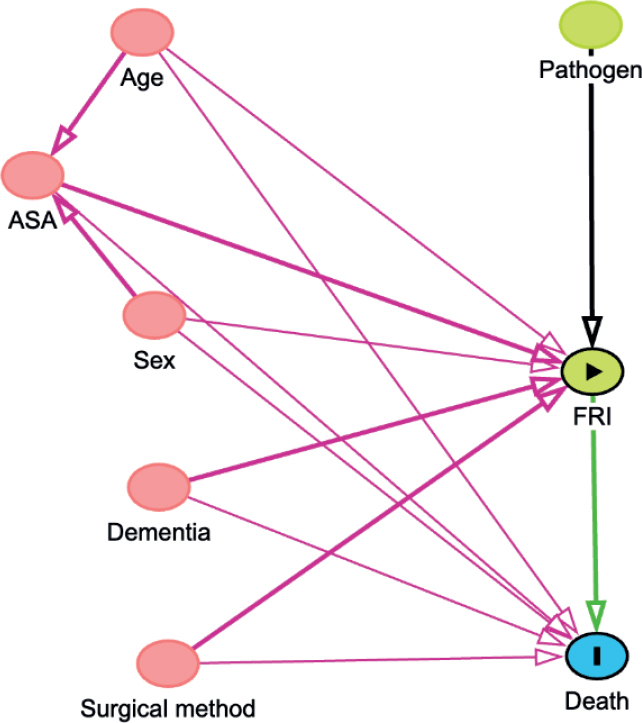
Illustration of relationships between variables in the study using a directed acyclic graph. The nodes represent death (outcome), FRI (exposure), ASA classification, age, dementia, pathogens, and sex. Arrows indicate directional relationships between variables. The graphical model was utilized to facilitate the selection of variables for adjustment in the Cox regression analysis.

Due to non-normal data distribution, medians with interquartile ranges (IQRs) were used to describe the age, time from surgery to the FRI, and length of antibiotic treatments. We examined crude mortality rates at key time intervals. Mortality was estimated with Kaplan–Meier (KM) survival analysis. Cox regression hazard ratios (HRs) with 95% confidence intervals (CIs) were calculated to assess mortality risk, with FRI as a time-dependent main variable, and adjustment for the potential confounders ASA, sex, dementia, injury age, and surgical method. The covariates adjusted for were selected following the compilation of a directed acyclic graph (Figure 2, see Appendix). The exposure status (i.e., an FRI) can change during the observation period. To accommodate this change an FRI was used as a time-varying explanatory variable, in contrast to a regular Cox regression analysis, where exposure is assumed to occur simultaneously for all patients. Initially, all patients were categorized as unexposed to an FRI. For patients who subsequently developed an FRI, their status in the analysis shifted at the point of diagnosis. We structured the dataset to track the timing of FRI onset, allowing patients to be analyzed within the non-FRI group until their FRI diagnosis, after which they contributed to the FRI group’s data [16]. Because of early crossing of KM curves (see “Results” and Figure 4), indicating a possible violation of the assumption of proportionality over time, Schoenfeld residuals were estimated to check whether the underlying assumption of the Cox regression analysis was fulfilled. No violation of the proportionality assumption was found. Statistical significance was set at P < 0.05. The dataset was analyzed using Rstudio software, Version 2023.06.0+421 [17].

**Figure 3:**
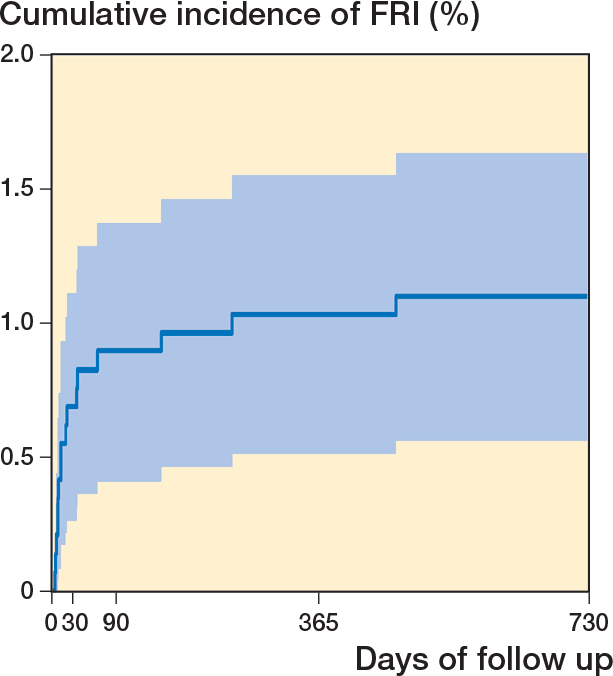
Kaplan–Meier estimates of cumulative incidence of patients developing an FRI (n = 16) within the study cohort (n = 1,455) over a 2-year period after hip fracture osteosynthesis.

**Figure 4:**
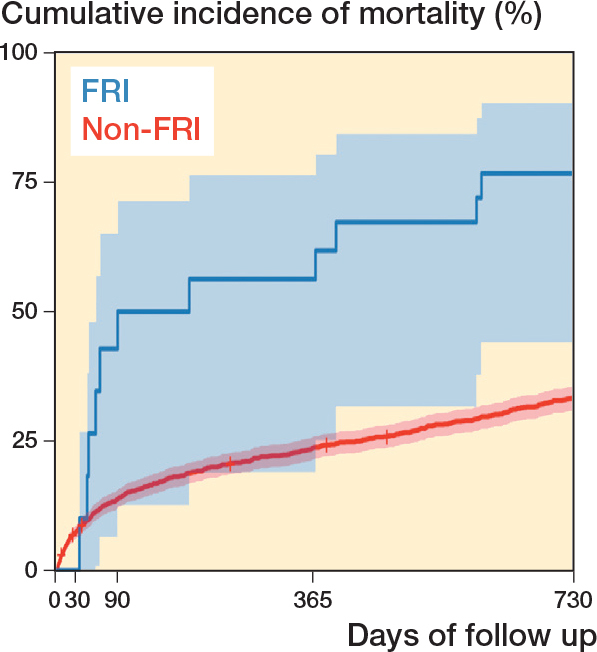
Kaplan–Meier estimates of cumulative incidence of mortality for the study cohort (n = 1,455) over a 2-year period after hip fracture osteosynthesis surgery, including 95% confidence intervals, for the non-FRI and FRI groups, with FRI as a time-dependent variable.

### Ethics, registration, data sharing plan, funding, use of AI, and disclosures

The study complied with the Declaration of Helsinki and was approved by the Swedish Ethical Review Authority (2021-04574 and 2021-06946-02). The datasets generated and analyzed during the current study are available from the corresponding author on reasonable request and in accordance with Swedish legislation. Data from the Swedish Fracture Register can be extracted from the Register Centre, Västra Götaland, after obtaining an approved ethical application. This work was supported by the research committee of Region Värmland (LIVFOU), ALF funding Region Örebro County.

The authors disclose that ChatGPT, GPT-4 (OpenAI) was used for spelling, grammar, and improving readability. The authors declare no competing interests. Complete disclosure of interest forms according to ICMJE are available on the article page, doi: 10.2340/17453674.2024.41980

## Results

### Participants

In the initial cohort of 2,536 patients with hip fractures, a total of 1,081 patients were excluded. Among these, 599 patients had undergone hemiarthroplasty and 338 had total hip arthroplasty. Another 144 patients were excluded either due to pathological fractures, non-surgical treatment, or because their surgical treatment, postoperative care, or follow-up was not performed in Uppsala. This resulted in a hip fracture group of 1,455 patients who were included in the analysis. In this group, a subset of 56 patient showed signs of wound infections or received antibiotic treatment, of which 16 were classified as an FRI according to definition (Figure 1).

### Incidence of FRI

The cumulative incidence of an FRI during the 2-year follow-up was 1.1% (16 of 1,455 patients). 2 of these patients were diagnosed after a reoperation because of primary malreduction. The cumulative incidence of FRIs throughout the study period is depicted in Figure 3.

### Characteristics of the study population

The median age within the FRI group was 83 years (IQR 71–87), and 50% were female. Among non-FRI patients, median age was 83 (IQR 75–89) years and 67% were female. The median time from surgery to FRI was 19 (IQR 11–38) days. In both the FRI and the non-FRI groups, most patients were classified as ASA III (69% and 60%, respectively). However, the proportion of ASA 4 patients was considerably higher in the FRI group (3 of 16) compared with the non-FRI group (6.4%) (Table 1).

**Table 1:** Demographic and clinical characteristics of the 1,455 hip fracture patients with and without fracture-related infection (FRI and non-FRI groups). Distribution as numbers (%) for sex, age groups, comorbidity based on the American Society for Anesthesiologists’ score (ASA 1–5), dementia, fracture type, and Arbeitsgemeinschaft für Osteosynthesefragen (AO) classification (2007), surgical method, and time to FRI diagnosis

Factor	Non-FRI group n = 1,439	FRI group n = 16
Sex		
Female	966 (67)	8
Male	473 (33)	8
Age at injury,		
median	83	83
IQR	75–89	70.5–86.5
Age groups		
< 60	64 (4.4)	1
60–75	301 (21)	4
> 75	1074 (75)	11
ASA score		
1	59 (4.1)	0
2	418 (29)	2
3	867 (60)	11
4	92 (6.4)	3
5	3 (0.2)	0
Dementia	383 (27)	6
Fracture type		
Intracapsular	425 (30)	3
Extracapsular	1,014 (70)	13
Location		
Neck of femur	425 (30)	3
Trochanteric	818 (57)	8
Subtrochanteric	196 (14)	5
AO classification		
MAO – 31 – A1	246 (17)	3
MAO – 31 – A2	568 (40)	6
MAO – 31 – A3	195 (14)	4
MAO – 31 – B1	310 (22)	2
MAO – 31 – B2	70 (4.9)	1
MAO – 31 – B3	41 (2.9)	–
MAO – 31 – C1	3 (0.2)	–
Not classified	6 (0.4)	–
Surgical method		
Intramedullary nail	602 (42)	8
Sliding hip screw	526 (37)	6
Screw/pin fixation	308 (21)	2
Other/combined	3 (0.2)	0
FRI diagnosis		
Early (< 2 weeks)	–	6
Delayed (2–10 weeks)	–	7
Late (> 10 weeks)	–	3

### Diagnosis, microbiology, and treatment

Most FRI cases (15 of 16) occurred within the first year post-surgery, with half of the group diagnosed within the first 3 weeks. The criterion of ≥ 2 positive tissue cultures was met for 12 of 16 patients. The remaining 4 patients had a fistula, purulent drainage from the wound or presence of pus during surgery. The prevalence of monomicrobial infection was highest, affecting 10 patients, followed by polymicrobial infections in 5 patients, and 1 patient with negative culture results.

*Staphylococcus aureus* was identified as the most prevalent pathogen, accounting for 10 of 15 FRI patients with positive cultures; all strains were susceptible to methicillin. Most (15 of 16) of FRI patients required at least 1 revision, and 9 needed 2 or more revision surgeries, 8 had total or partial hardware removal, and 4 underwent final revision to the prosthesis. 10% (139 of 1,455) of the patients in the entire cohort underwent reoperation for various reasons. Among these, 8% (11 of 139) of patients had a non-union (none of these were identified as having an FRI), and 10% (14 of 139) of patients experienced mechanical failure, with 1 being diagnosed with an FRI. Overall median antibiotic treatment for the surviving FRI patients (7 patients died during antibiotic treatment) was 114 days (IQR 67–128), consisting of 18 days (IQR 11–23) with intravenous antibiotic therapy and 92 (IQR 46–112) with oral antibiotic therapy.

### Mortality

The 2-year crude mortality rate was 11 of 16 (69%) in the FRI group versus 32.7% in the non-FRI group (Table 2). 8 of 11 patients who died within 2 years were diagnosed with FRI caused by *S. aureus*. The KM analysis also depicted a higher cumulative incidence of mortality in the FRI group (Figure 4). Moreover, the Cox regression analysis, with FRI as a time-dependent variable, resulted in an HR of 4.1 (CI 2.3–7.5) for FRI patients compared with non-FRI patients. After adjusting for ASA, sex, age, dementia, and surgical method, the HR was 3.5 (CI 1.9–6.4) for FRI patients (Table 3).

**Table 2:** Cumulative all-cause mortality, numbers (%), for 1,455 hip fracture patients with and without fracture-related infection (FRI and non-FRI groups) within 30 days, 90 days, 1 year, and 2 years after initial surgical treatment

Time	Non-FRI group n = 1,439	FRI n = 16
30 days	104 (7.2)	0
90 days	194 (13.5)	6
1 year	333 (23.1)	7
2 years	471 (32.7)	11

**Table 3:** Association between fracture-related infection (FRI) and mortality assessed by Cox regression analysis. Hazard ratios (HR) and 95% confidence intervals (CI) (unadjusted and adjusted for comorbidity based on the American Society for Anesthesiologists’ score (ASA 1–5), dementia, sex, age, and surgical method, with FRI as a time-dependent and key predictor variable

Variable	HR (CI)
Unadjusted	
FRI: time-dependent	4.1 (2.3–7.5)
Adjusted	
FRI: time-dependent	3.5 (1.9–6.4)
ASA 2	2.7 (0.7–11)
ASA 3	5.7 (1.4–23)
ASA 4	12.6 (3.1–52)
ASA 5	47.4 (6.6–341)
Dementia	1.4 (1.2–1.7)
Male sex	1.6 (1.3–1.9)
Age at injury	1.1 (1.1–1.1)
Sliding hip screw	1.0 (0.9–1.3)
Screw/pin fixation	1.0 (0.8–1.3)

Age at injury quantifies the impact of each additional year on the outcome. ASA 1 is the reference category for comparing the relative risks associated with other ASA classifications and intramedullary nail is the reference for surgical methods.

## Discussion

Our primary aim was to assess the association between FRIs and mortality after osteosynthesis for hip fracture, excluding arthroplasties. We found a significant association between FRIs and increased mortality after osteosynthesis for hip fracture In addition, we showed a cumulative incidence presented in our study, which was 1.1% for FRIs at a 2-year follow-up in hip fractures treated with osteosynthesis, consistent with the contemporary criteria for an FRI. This is in line with the historically reported incidence of FRI, ranging from 1–2% for closed fractures [18], and also corresponds with previous findings showing that the incidence of deep infection after hip fracture surgery can vary between 0.72 and 2.5% [3-6]. However, previous research has incorporated a combination of surgical techniques (such as osteosyntheses and prostheses) or used alternative definitions of a postoperative infection. This may have impeded the comprehension of FRIs in hip fractures treated with osteosynthesis [9-11]. Even though both FRIs and PJIs in a mixed hip fracture cohort arise from treating a fracture with implants surgically, the clinical manifestations, diagnostic approaches, and especially treatment regimens differ considerably between the two. This approach can potentially yield discrepant conclusions, as PJIs and FRIs after osteosynthesis represent separate entities [4]. A study by Wang et al. used the current FRI criteria and reported an incidence of 1.5% [19]. This study included multiple fracture sites and open and closed fractures, with more than half of the reported FRI cases following fractures distal to the knee. Studies focusing on specific anatomical fracture sites are warranted to generalize results and improve future preventive strategies. We believe the precision of our approach strengthens our incidence data as we focused solely on the osteosyntheses of hip fractures and strictly followed the FRI definition.

### Demographics

The FRI group showed an age distribution similar to the broader hip fracture population while demonstrating an even sex distribution, contrasting with the predominance of females observed in the non-FRI group. Due to the relatively small FRI group, further investigation is warranted to gain a better understanding of sex-related risk factors in developing FRIs. Other studies have shown a potential male predominance in FRI patients [20].

A clear bias in cohorts of mixed fracture locations is the increased risk of FRIs in open fractures, which are more common in younger men. In our FRI group the median age was high (83 years), and there were no open fractures, minimizing such a potential bias. We found that most patients in the FRI and non-FRI groups were categorized as ASA 3, which corresponds to patients with severe systemic disease. This observation underscores the complex health conditions of patients undergoing hip fracture surgery, potentially affecting their susceptibility to complications such as an FRI. This correlation has been investigated thoroughly for surgical site infections (SSIs) after hip fracture surgery [9,10], but not in correlation to the more strict, robust, and consistent FRI definition for hip fractures treated with osteosynthesis [15]. Of note, we observed a higher proportion of ASA 4 patients in the FRI group, indicating a potential association between severe systemic disease and an increased risk of an FRI. Previous reports also suggest an association between higher ASA scores and an increased likelihood of complications after hip fracture surgery [21]. Yet, ASA is mere a proxy variable of health and does not capture the patient’s general status with high precision or in detail.

### Diagnosis, microbiology, and treatment

All FRI patients were diagnosed through confirmatory criteria. None of the patients were diagnosed through culturing sonication fluid. Although this method has shown promising results, especially for low-grade infections, it has not yet been widely adopted in Sweden [22]. The majority of FRI cases occurred within the first year after surgery, confirming previous literature [23]. As in other studies investigating the etiology of FRIs, *S. aureus* was the most prevalent causative pathogen [19,24]. While *S. aureus* is known for its virulence, patients can exhibit relatively mild symptoms even in the presence of *S. aureus* bacteremia, even though it carries a mortality rate of 20–30% [25]. This possible discrepancy between clinical presentation and mortality risk, in combination with the high prevalence of *S. aureus* infections in patients with FRI, complicates the treatment. Over half of the patients in our FRI group with *S. aureus* infections lacked blood cultures. The question arises as to whether implementing more rigorous diagnostic methods for this patient group (such as routine blood cultures) might improve outcomes. The substantial proportion of FRI patients requiring revision surgery confirms previous findings, underscoring the severity of the condition [26].

Half of the patients in the FRI group underwent some form of hardware removal. In a retrospective study by Halonen et al., total implant removal was not generally necessary for trochanteric hip fractures treated with intramedullary nails in cases of deep SSI [27]. However, 36% (4 out of 15 patients) required some form of partial hardware removal. Further research is needed to determine how translatable these findings are to patients diagnosed according to FRI criteria. Although revision surgery is often a necessity for cure according to FRI treatment guidelines, it may not be the optimal treatment for every patient [28]. In some cases, the best path to a tolerable quality of life might be to avoid surgery. This decision can be influenced by the patient’s overall health status or comorbidities that renders surgical treatment ineligible. The combination of advanced age, high comorbidity, a clinical scenario where surgery is deemed unfit and/or a low-grade, low-virulent FRI might make lifelong suppressive antibiotic therapy a tolerable and more adequate alternative, even if it does not result in a cure in terms of infection eradication per se.

The median antibiotic treatment course in our FRI group is partially consistent with current international recommendations and treatment guidelines [29]. An incomplete adherence could be due to the fact that the FRI definition and subsequent recommendations were introduced relatively late in the inclusion period of the cohort, leading to a change in the Swedish national guidelines by the end of 2018.

### Mortality

The FRI group had a higher mortality rate at 2 years compared with the non-FRI group. This finding is in line with previous studies highlighting the adverse impact of SSIs on survival in patients with hip fractures [9]. Our findings align to some extent with those of Wong et al., who reported a significant association between FRIs and increased mortality risk, regardless of the infection’s depth or temporality [12]. The majority of deaths in our FRI group were in patients with *S. aureus* infections (8 of 11). Given the small size of our group, extensive statistical analysis is constrained, and such quantifications should be approached with caution. The observed descriptive patterns suggest that pathogen-specific traits, especially for virulent bacteria such as *S. aureus*, may contribute to mortality in FRI. Age and preoperative comorbidities correlate with postoperative complications and mortality [30]. A serious musculoskeletal infection adds to the burden of these already vulnerable patients, and treatment choices and monitoring are of utmost importance. For example, nephrotoxic antibiotics can trigger acute renal failure in frail elderly patients, which significantly increases the risk of mortality [31].

### Strengths and limitations

***Strengths.*** This is a single-center design, ensuring a uniform approach to patient management and data collection, thereby enhancing the internal validity of our findings. It may, however, limit generalization of the results to other centers. We had a standardized and strict definition of FRI which increases the comparability of our results with those of previous and future FRI studies.

***Limitations.*** The retrospective nature of our study introduces inherent risks, including the possibility of missing data, potential selection bias, and unmeasured confounders. The FRI group was small, impacting the power of our statistical analyses and limiting the generalizability of the findings. The 16 patients in the FRI group were identified from a group of 56 patients who presented with postoperative complications at the fracture site or received postoperative antibiotic treatment. Hence, the remaining 40 patients did not meet the FRI criteria. If there were wound reactions interpreted as infectious signs, these reactions could have been caused by other non-specific postoperative reactions, e.g., to sutures or staples. Additionally, the postoperative antibiotic treatment might have been merely prophylactic or addressing conditions unrelated to a potential FRI. The process used to select this subset was only partially standardized. Nevertheless, we believe that the incidence of undetected FRI cases is negligible, considering that the selection process likely favored sensitivity over specificity.

Determining the true incidence of FRI can be challenging. Infection has been identified as a cause of non-unions in 30% of femoral and tibial diaphyseal fractures following intramedullary nailing [32]. In our cohort, deep tissue cultures were not routinely taken in cases of non-union or mechanical failure during 2015–2019, which accounted for 18% (25 of 139) of the reoperations in the cohort. This may potentially lead to underestimation of the FRI incidence. Furthermore, FRI can often result in an acute onset of illness, which can lead to rapid death at home. Since autopsies are rarely performed, the cause of death might be misinterpreted, potentially overlooking an FRI that led to sepsis and death.

### Conclusion

Our study shows a significant association between FRIs and increased postoperative mortality after hip fracture osteosynthesis surgery, even after adjusting for relevant confounders.

In perspective, our findings also underline the severe consequences of FRIs, including prolonged treatment regimens, and suggest a potential link between advanced systemic diseases and an increased risk of an FRI. The findings from this study contribute to the broader understanding of FRIs in hip fractures, supporting health care professionals in optimizing prevention and treatment protocols as well as patient care strategies.

## Notes

All authors contributed to the design of the study. PK and AB performed the data analysis. PK wrote the manuscript draft. AB, ST, PF, NPH, and OW made critical revisions.

Handling co-editors: Taco Gosens

*Acta* thanks Jan-Erik Gjertsen, Pieter Leliefeld, Rene Mihalič, and Cornelis Leendert Pieter van de Ree for help with peer review of this manuscript.
